# Factors associated with lack of tracheal sealing by a cuff inflated to more than 30 cmH_2_O during mechanical ventilation: A cross-sectional study

**DOI:** 10.12669/pjms.39.2.5672

**Published:** 2023

**Authors:** Hong-Lei Wu, Hai-Yan Shi, Jia-hai Shi, Wang-Qin Shen

**Affiliations:** 1 Hong-Lei Wu, BN., Nursing Department, Affiliated Hospital of Nantong University, Nantong University, Jiangsu Province, 226001, China; 2 Hai-Yan Shi, MN., Nursing Department, The People’s Hospital of Rugao, and Affiliated Rugao Hospital of Nantong University, Nantong City, Jiangsu Province, 226001, China; 3 Jia-hai Shi, MD., Department of Cardiothoracic Surgery, Affiliated Hospital of Nantong University, Jiangsu Province, 226001, China; 4 Wang-Qin Shen, MD., Nursing Department, Nantong University, Jiangsu Province, 226001, China

**Keywords:** Cuff pressure, Unsealed trachea, Cuff diameter

## Abstract

**Objectives:**

The cuff pressures > 30 cmH_2_O may create a seal in the trachea. The objective of this study was to identify risk factors associated with lack of tracheal sealing by an endotracheal cuff inflated to > 30 cmH_2_O in patients undergoing mechanical ventilation.

**Methods:**

This prospective cross-sectional study was conducted from 2019 to 2020 in the cardiothoracic intensive care unit and respiratory medical care unit of a Hospital in Nantong, China. Patients aged >16 years undergoing cardiothoracic surgery with mechanical ventilation using endotracheal intubation were included. Patient characteristics and ventilator parameters were analyzed. Cuff pressure was maintained with the minimum leak technique (MLT) and measured with a cuff pressure gauge. Cuff pressure was measured for 30 seconds when ventilation was accompanied by no leak, simultaneously detected by the ventilator or auscultation with a stethoscope.

**Result:**

Of 352 patients undergoing mechanical ventilation, 51 patients (14.5%) had a cuff pressure of >30 cmH_2_O. Multivariable analysis showed that cuff manufacturer (Guangzhou Weili) and nasal endotracheal intubation significantly increased the risk of an unsealed trachea. Peak inspiratory pressure, cuff diameter and male sex had a strong inverse association with an unsealed trachea.

**Conclusions:**

These findings suggest that an endotracheal cuff pressure of 20 to 30 cmH_2_O is adequate for most patients, but lack of a tracheal seal still occurs in a small number of people. An unsealed trachea is most likely because cuff and tracheal diameters do not match.

***Clinical Trial Registration:*** http://www.chictr.org.cn/index.aspx Unique identifier: ChiCTR-COC-15006459.

## INTRODUCTION

Endotracheal intubation is a common medical procedure that is used in emergency situations.^1^ Some endotracheal tubes have a high-volume, low-pressure cuff that creates a seal between the endotracheal tube and the trachea, preventing aspiration of fluids and pathogens from the pharynx to the lungs and ventilation leaks.^2^-^5^ Consensus suggests that cuff pressure in endotracheal tubes should range from 20 to 30 cmH_2_O.^4^,^6^-^8^ Excessively high or low cuff pressures have been associated with complications such as tracheal stenosis, leaking of tidal volume, micro-aspiration of secretions, and ventilator-associated pneumonia.^4^,^9^,^10^ In 1984, Seegobin and van Hasselt^5^ analyzed the relationship between cuff pressure and tracheal mucosal blood flow. Findings showed that mucosal capillary blood flow was impaired when cuff pressure exceeded 30 cmH_2_O, and mucosal capillary blood flow was completely obstructed when cuff pressure exceeded 50cm H_2_O.

Evidence-based guidelines recommend that cuff pressure is maintained between 20 and 30cm H_2_O. However, in clinical practice cuff pressures > 30 cmH2O^2^-^4^ may be required to create a seal in the trachea, which may compromise perfusion and the integrity of the tracheal mucosa. Previous reports indicate that cuff pressure may be altered by duration of intubation, patient body temperature and movements, and positive-pressure ventilation.^11^ In most cases, selection of a cuff is based on a patient’s weight and sex. The most commonly utilized cuffs in adults are appropriately sized to provide a seal through tracheal mucosal contact.^12^ In a small number of patients, cuff size may be unsuitable.

This variability is reflected in a large number of studies, and there is no consensus on optimal cuff pressure targets.^13^,^14^ Most institutions utilize cuff pressures of 20 to 30 cmH_2_O; however, it is unclear why this may not achieve proper sealing between the trachea and the cuff wall in all patients. If cuff pressure is managed in strict accordance with guideline recommendations, leakage around the cuff may impede ventilation and lead to ventilator-associated pneumonia. The objective of this study was to identify risk factors associated with lack of tracheal sealing by a cuff inflated to > 30 cmH_2_O in patients undergoing mechanical ventilation. In this cross-sectional study, we collected data concerning current clinical practice at an institutional level rather than personal views or opinions. Findings will ensure safe cuff pressures are used in patients undergoing mechanical ventilation.

## METHODS

This single-center, prospective cohort study was performed in the cardiothoracic intensive care unit and respiratory medical care unit of a hospital in Nantong, China between June 2019 and October 2020. This study was approved by the Medicine Human Studies of Nantong University affiliated hospital (2015--108) on May 21, 2015. The study was registered with the Chinese Clinical Trial Registry (ChiCTR-COC-15006459) on 29 May 2015, http://www.chictr.org.cn/index.aspx.

### Inclusion criteria:

Age ≥16 years;
Willingly provided informed consent;
Had current anthropometric data. Patients with unstable vital signs or massive pleural effusion were excluded.

The following factors were examined to determine their influence on the ability of an endotracheal cuff to maintain a seal within an airway: cuff diameter, duration of time required for endotracheal intubation, timing of intubation, duration of sedation, number of times the patient was repositioned, number of times suctioning was performed,^15^ head position^16^ sex,^17^ age,^18^ and weight and height.^19^

### Instrument and equipment:

AVEA ventilator systems (Vyaire Medical, Mettawa, IL, USA) were used in this study. The mode of mechanical ventilation was determined by the physician. Other equipment included reinforced endotracheal tubes (Guangzhou Weili Medical Equipment Co., Ltd., Guangzhou, China and Jiangxi Galanz Medical Equipment Co., Ltd., Jiangxi, China), ordinary endotracheal tubes (Guangzhou Weili Medical Equipment Co., Ltd.), tracheotomy cannula (Guangzhou Weili Medical Equipment Co.Ltd), cuff pressure gauges (Hangzhou Ranran Trade Co., Ltd., Hangzhou, China), stethoscopes (Beijing Hausheng Technology and Trade Co., Ltd., Beijing, China), and retractable tubes with a 1.5-m suction loop (Intesec Medical Devices Co., Ltd., Changzhou, China).

### Patient Assessment:

Patients were evaluated by a nurse from the ICU who informed them orally about the purpose and risks of this study. Written, informed consent was obtained from all patients or their legal representatives. Patient age (years), sex, height (m), weight (kg), and body mass index (kg/m^2^) were obtained from the hospital information system. Ventilator parameters were set by physicians according to the patients’ weight and clinical characteristics. Mechanical ventilation was initiated in patients with stable vital signs who required assisted spontaneous breathing after two hours in the supine position. Sedatives were administered to patients with strong spontaneous respiratory effort. The method of endotracheal intubation (oral, nasal cavity, or tracheotomy), cuff diameter and cuff manufacturer were recorded. Tidal volume (VT), peak inspiratory pressure (PIP) and respiratory rate (RR) were derived from the ventilator.

### Cuff Pressure:

Cuff pressure management was performed by one of four nurses with at least five years of work experience in the ICU. Each nurse used the same brand of cuff pressure gauge (Hangzhou Ranran Trade Co., Ltd., Hangzhou, China). Cuff pressure was maintained with the minimum leak technique (MLT).^13^ Negative pressure and a sputum suction tube were used to clear patient’s oral and nasal secretions. Cuff pressure was measured for 30 seconds when ventilation was accompanied by no leak. A stethoscope was used to confirm there was no audible air leak. Cuff pressure was stratified as ≤30 cmH_2_O (sealed trachea) or >30 cmH_2_O (unsealed trachea).

### Statistical Analysis:

Statistical analyses were conducted using SPSS version 20 (IBM Corp., Armonk, NY, USA) and Prism 8 (GraphPad, San Diego, CA, USA). Data for patients with no cuff pressure values were excluded from the analysis. Descriptive statistics were used to compare patient baseline characteristics and outcomes. Univariate chi-square tests and t tests were used to compare categorical variables and continuous variables, respectively. P < 0.05 was deemed significant. Multivariate logistic regression analysis was used to evaluate the impact of the variables identified as primary risk factors for tracheal leakage at a cuff pressure > 30 cmH_2_O (TLA30). Odds ratios (ORs) and corresponding 95% confidence intervals (CI) were calculated after adjusting for the effects of potential confounding variables.

## RESULTS

**Fig. 1:**
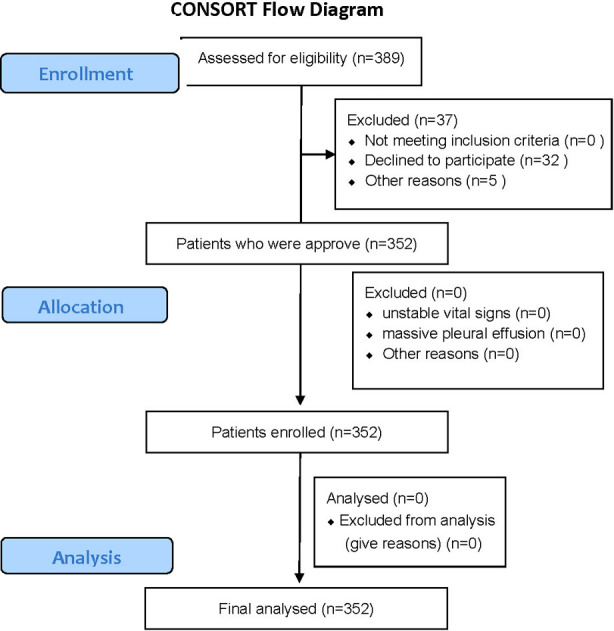
CONSORT flow diagram.

**Fig.2:**
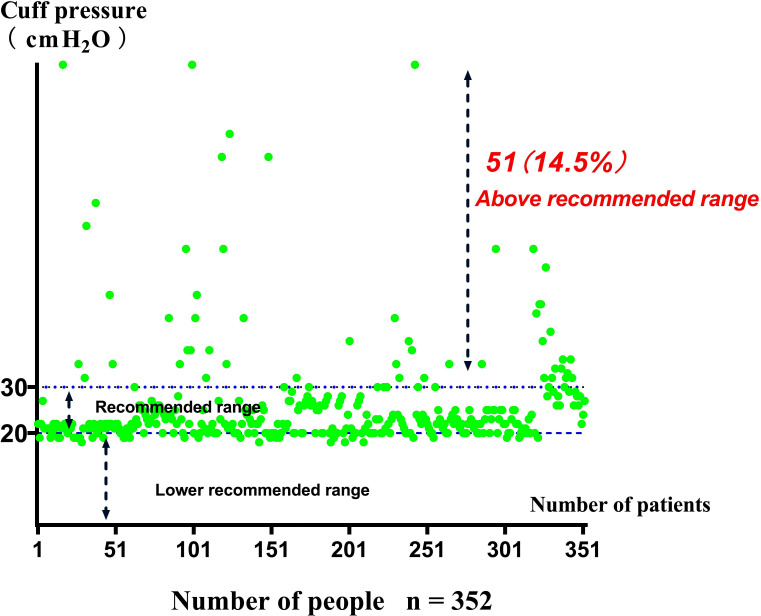
Incidence of cuff pressure >30 cmH_2_O in patients undergoing mechanical ventilation.

A total of 389 patients undergoing mechanical ventilation were eligible for this study, 37 patients met the exclusion criteria, and 352 patients agreed to participate (Fig.1). Among the 352 participants, 312 (88.6%) patients were receiving treatment in the cardiothoracic intensive care unit, and 40 (11.4%) patients were receiving treatment in the respiratory medical care unit. Mean age of patients was 62.87 ± 13.41 years, 228 (64.8%) patients were aged ≥60 years, and more than half of the patients (69.6%) were male. Patient’s mean height was 1.658 ± 0.0812 m, and mean body mass index was 23.51 ± 3.397 kg/m^2^. Most patients underwent oral endotracheal intubation (92.3%), nasal endotracheal intubation was performed in 4.6% of patients, and tracheotomy was performed in 3.1% of patients. Among the ventilator parameters, mean V_T_ was 577.4 ± 95.21 mL and mean RR was 13.61 ± 2.76 breaths/min (Table-I).A total of 51 patients had a cuff pressure of >30 cmH_2_O (48 men and three women), which occurred at an incidence of 14.5% (95% CI: 10.8–18.2%) (Fig.2).

**Table-I:** Patients demographic and clinical characteristics.

***Parameter***	
Number of subjects (*n*)	352
Age (y)	62.87 ± 13.41
Weight (kg)	64.75 ± 11.34
Height (m)	1.658 ± 0.08122
BMI	23.51 ± 3.397
Tidal volume (ml)	577.4 ± 95.21
Respiratory rate (n)	13.61 ± 2.76
***Ward(n)***	
CSICU	312(88.6%)
RICU	40(11.4%)
***Surgery (n)***	
Yes	311(88.4%)
No	41(11.6%)
***Sex (n)***	
Men	245(69.6%)
Women	107(30.4%)
***Methods of endotracheal intubation (n)***	
Oral type	325(92.3%)
Nasal type	16(4.6%)
Tracheotomy type	11(3.1%)

CSICU: Cardiothoracic Intensive Care Unit; RICU: Respiratory Medical Care Unit.

**Fig.3:**
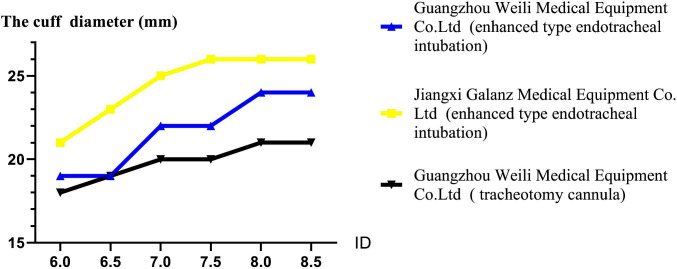
Cuff diameters of endotracheal tubes from different manufacturers (ID: inner diameter.

The cuff diameter of the endotracheal tube manufactured by Jiangxi Galanz Medical Equipment Co., Ltd was significantly larger than the cuff diameter of the endotracheal tube manufactured by Guangzhou Weili Medical Equipment Co. Ltd or the tracheotomy cannula manufactured by Guangzhou Weili Medical Equipment (Fig.3).

Unadjusted and adjusted binary logistic regression analyses identified several independent risk factors for TLA30 during mechanical ventilation, including male sex, cuff diameter, and PIP (Table-II and III). Male sex (OR, 18.513; 95% CI, 4.897–69.982; p < 0.0001), oral endotracheal intubation (OR, 19.694; 95% CI, 1.093–354.792; p = 0.043), nasal endotracheal intubation (OR, 66.995; 95% CI, 4.033–1112.895; p = 0.003), peak inspiratory pressure (OR, 1.138; 95% CI, 1.057–1.223; p < 0.0001), cuff diameter (OR, 0.394; 95% CI, 0.251–0.620; p < 0.0001), cuff manufacturer GZW (OR, 0.092; 95% CI, 0.02–0.427; p = 0.002), and PIP (OR, 1.138; 95% CI, 1.059–1.223; p < 0.0001), were risk factors for TLA30 during mechanical ventilation (Table-IV).

**Table-II:** Univariate analysis of risk factors for TLA30.

Parameter	Unsealed Trachea Group	Sealed Trachea Group	x2/t	P
Gender (M/F)	48/3	197/104	16.946	0.0001*
Age(year)	69.14±11.94	61.81±13.37	3.673	0.0003*
Weight (Kg)	64.78±11.74	64.74±11.29	0.0243	0.9806
Height(cm)	168.7±6.723	165.2±8.238	2.874	0.0043*
BMI	22.73±3.831	23.64±3.307	1.768	0.0780
Operation(yes/no)	32/19	279/22	35.85	0.0001*
EIIP(OEI/ NEI/TC)	36/8/7	289/8/4	40.79	0.0001*
PIP(cmH2O)	23.49±7.103	20.19±3.725	4.988	0.0001*
TDOC(mm)	23.18±1.977	24.18±1.667	3.850	0.0001*
Factory(GZW1/ GME/ GZW2)	24/20/7	160/137/4	22.144	0.0001*
Tidal volume(ml)	564.9±91.50	579.6±95.81	1.016	0.3104
Respiratory rate	15.53±4.293	13.20±2.082	6.589	0.0001*
Temperature	37.54±0.9278	37.55±0.8979	0.0806	0.9358

M: Male; F: Female; BMI: Body Mass Index; EIIP: Endotracheal intubation implantation pathway OEI: Oral endotracheal intubation; NEI: Nasal endotracheal intubation; TC: Tracheotomy cannula; PIP: Peak inspiratory pressure; TDOC: The diameter of cuff; GZW1: Guangzhou Weili Medical Equipment Co.Ltd (enhanced type endotracheal intubation); GME: Jiangxi Galanz Medical Equipment Co. Ltd (enhanced type endotracheal intubation); GZW2: Guangzhou Weili Medical Equipment Co.Ltd (tracheotomy cannula); ID: inner diameter.

**Table-III:** Unadjusted logistic regression analysis of independent risk factors for TLA30.

Parameter	Odds ratio	B	SE	P	95% CI
Gender (Male)	11.313	2.426	0.749	0.001*	2.607 - 49.084
Age	1.016	0.016	0.016	0.299	0.986 -1.048
Height	1.026	0.026	0.027	0.351	0.972 -1.082
***Operation***					
Y	0.599	-0.513	0.728	0.481	0.144 – 2.495
***Endotracheal intubation implantation pathway***					
OEI	16.369	2.795	1.495	0.062*	0.874 – 306.758
NEI	37.483	3.624	1.530	0.018*	1.867 – 752.641
Peak inspiratory pressure	1.119	0.113	0.039	0.004*	1.037 - 1.209
The diameter of cuff	0.425	-0.855	0.236	<0.0001*	0.268 - 0.676
***Cuff manufacturer***					
GZW	0.168	-1.786	0.852	0.036*	0.032 - 0.891
Respiratory rate	1.077	0.075	0.070	0.286	0.940 - 1.235
constant	547.168	6.305	5.965	0.291	

OEI: oral endotracheal intubation; NEI: Nasal endotracheal intubation; GZW: Guangzhou Weili

**Table-IV:** Adjusted logistic regression analysis of independent risk factors for TLA30.

Parameter	Odds ratio	B	SE	P	95% CI
Gender (Male)	18.513	2.918	0.678	<0.0001*	4.897 - 69.982
***Endotracheal intubation implantation pathway***					
OEI	19.694	2.980	1.475	0.043*	1.093 - 354.792
NEI	66.995	4.205	1.434	0.003*	4.033 - 1112.895
Peak inspiratory pressure	1.138	0.129	0.037	<0.0001*	1.059 - 1.223
The diameter of cuff	0.394	-0.931	0.231	<0.0001*	0.251 - 0.620
***Cuff manufacturer***					
GZW	0.092	-2.389	0.784	0.002*	0.02 - 0.427
constant	700740.651	13.460	8.288	0.004	

OEI: oral endotracheal intubation; NEI: Nasal endotracheal intubation; GZW: Guangzhou Weili.

## DISCUSSION

Evidence-based guidelines recommend that endotracheal cuff pressure is maintained between 20 and 30 cmH_2_O during surgery and mechanical ventilation.^7^ However, findings from the present study imply that a cuff pressure of 20 to 30 cmH_2_O is not ideal for every patient. The optimal cuff pressure should ensure adequate ventilation while concurrently avoiding tissue ischemia, ulceration, and necrosis of the tracheal wall and air leak.^20^ Preserving cuff pressure within a desirable range is challenging because cuff pressure may be influenced by various patient-related factors, environmental conditions, and medical interventions.^16^ Our results indicate that TLA30 during mechanical ventilation is associated with patient sex, method of endotracheal intubation, cuff diameter, cuff manufacturer, and PIP. Within healthcare organizations, the effects of method of endotracheal intubation and cuff manufacturer on TLA30 remain constant as they are determined by protocols and budgets. The factors associated with TLA30 that have most relevance for the physician include patient sex, cuff diameter, and PIP.

### Sex as a risk factor for TLA30:

Our analyses showed that male sex was associated with a >10-times higher risk of TLA30 than female sex (OR, 11.042; 95% CI, 2.493–48.904; p = 0.002). The findings are consistent with previous reports. In an observational cross-sectional study in a tertiary metropolitan intensive care unit that assessed the relationship between the MLT and cuff manometry, univariate analysis showed female patients required lower volumes in their cuffs and had smaller endotracheal tubes than males.^21^ In a retrospective study that aimed to acquire normative data on central airway dimensions on chest CT scans in the pediatric population, a mixed-effects model showed male sex was a significant predictor of a larger diameter of the trachea, right main bronchi, and left main bronchi in boys > 14 years of age.^18^ However, when computed tomography was used to determine tracheal lengths, diameters and cross-sectional areas, multiple regression analyses that used sex as a covariate showed no differences in tracheal dimensions between males and females after the effect of height was eliminated. The disparate findings among the present study and previous reports may arise from differences in the demographic and clinical characteristics of the study populations. In the present study, the population comprised more males (n=245; 69.6%) than females (n=107; 30.4%), and we were unable to assess to the effects of various disorders, such as inflammatory or traumatic conditions, tumors and infections, which may cause tracheal stenosis or tracheomalacia.^22^

### Cuff diameter as a risk factor for TLA30:

**Fig.4:**
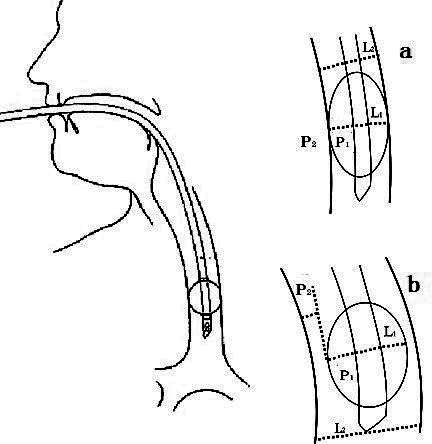
Schematic showing the relationship between endotracheal cuff diameter and tracheal diameter in patients undergoing mechanical ventilation. (a) Matching of cuff and tracheal diameters (L1 = L2). (b) Lack of matching between cuff and tracheal diameters (L1 < L2)

It is essential to carefully match the diameter of an endotracheal cuff to the diameter of the trachea. A cuff that is too large for the trachea may cause pressure necrosis of the tracheal soft tissues,^23^ while a cuff that is too small may result in insufficient sealing of the trachea. In clinical practice, there are no consensus criteria that facilitate close matching between the diameters of trachea and cuff. Findings from the present study indicate that the pressure within the cuff may not equal the pressure of the cuff on the tracheal mucosa. Assuming a standardized endotracheal tube cuff pressure of 30 cmH_2_O, if cuff and tracheal diameters match (L_1_ = L_2_), the pressure within the cuff will equal the pressure of the cuff on the tracheal mucosa (P_1_ = P_2_); however, if cuff and tracheal diameters do not match (L_1_ < L_2_) the cuff may exert pressure > 30 cmH_2_O (P_1_ > P_2_) (Fig.4).^7^,^24^

### Method of endotracheal intubation as a risk factor TLA30:

Available intubation techniques include oral endotracheal intubation, nasotracheal intubation, and tracheostomy. Findings from the present study suggest that cuff diameter for a given model of endotracheal tube varies across manufacturers. In clinical practice when the patient’s nasal cavity is small, the anesthesiologist may select a small endotracheal tube for nasotracheal intubation. There is the probability that the cuff size may be smaller than the patient’s airway diameter. Consequently, patients with transnasal tracheal intubation may require a higher cuff pressure to seal the trachea. In clinical practice, medical staff are unaware of the factors that influence TLA30. In this case, cuff and tracheal diameters may not match (L_1_ < L_2_ and P_1_ > P_2_).

### Limitations of the study:

First, it was a cross-sectional study, and the small sample size may have affected the results. Second, its generalizability to other areas of China may be limited because all participants were from a single hospital. Third, we were unable to analyze all parameters recommended for cuff assessment because of the difficulty of obtaining these data from patients undergoing mechanical ventilation. Finally, our study population mainly comprised patients who underwent orotracheal intubation (92.3%).

## CONCLUSION

The present study indicates that an endotracheal cuff pressure of 20 to 30 cmH_2_O is adequate for most patients but may result in insufficient sealing of the trachea in a small number of patients. There is a need for comprehensive criteria on which to base target cuff pressure. These should include patient anthropometric and clinical data and specifications on the design and size of the endotracheal tube.

### Author contributions:

**HLW**: Wrote the manuscript, collected data, primary research/study designer.

**HYS**: Collected data, Analyzed data, primary research.

**JHS**: Offered guidance on study design and manuscript.

**WQS**: Analyzed data, Primary research/study designer, Accountable for the accuracy and integrity of the work.
